# Health information management of older, multimorbid patients in German primary care: feasibility and first results of the outcome measures of a cluster-randomised controlled pilot trial – HYPERION-TransCare

**DOI:** 10.1186/s12875-025-02774-5

**Published:** 2025-04-05

**Authors:** Astrid-Alexandra Klein, Maria Hanf, Truc Sophia Dinh, Franziska Brosse, Jenny Petermann, Steve Piller, Sylvia Schulz-Rothe, Lena Schäfer, Karola Mergenthal, Hanna M. Seidling, Sophia Klasing, Nina Timmesfeld, Bahareh Yousefi, Marjan van den Akker, Karen Voigt

**Affiliations:** 1 Department of General Practice, Faculty of Medicine and University Hospital Carl Gustav Carus, TUD Dresden University of Technology, Fetscherstraße 74, Dresden, 01307 Germany; 2 Institute of General Practice, Goethe University Frankfurt, Theodor-Stern-Kai 7, Frankfurt Am Main, Germany; 3 Institute of General Practice, Goethe University Frankfurt, Theodor-Stern-Kai 7, Frankfurt Am Main, 60590 Germany; 4 Cooperation Unit Clinical Pharmacy, Internal Medicine IX - Department of Clinical Pharmacology and Pharmacoepidemiology, Medical Faculty Heidelberg, Heidelberg University Hospital, Heidelberg University, Im Neuenheimer Feld 410, Heidelberg, 69120 Germany; 5 Department of Medical Informatics, Biometry and Epidemiology, Ruhr University Bochum, Universitätsstraße 105, Bochum, 44789 Germany; 6 Academic Center for General Practice, Department of Public Health and Primary Care, KU Leuven, Louvain, Belgium; 7 Department of Family Medicine, School CAPHRI, Maastricht University, Maastricht, the Netherlands

**Keywords:** Polypharmacy, Multimorbidity, General practice, Cluster-randomised controlled trial, Health information management, Feasibility study, Complex intervention, Health literacy, Combined endpoint

## Abstract

**Background:**

Availability of information at the interface of outpatient and inpatient care remains an important and inadequately resolved issue in Germany and beyond. As a vulnerable group with complex care needs, older patients, mostly multimorbid, are especially affected by the consequences. This trial tested the feasibility and implementability of a complex intervention which aimed at improving the availability of health information among patients and different healthcare providers.

**Methods:**

The prospective two-arm blinded pilot cRCT was accompanied by an explorative mixed-methods process evaluation. Over a period of six months in 2022, general practitioner (GP) practices in Germany with patients (≥ 65, multimorbid, polypharmacy) participated in testing the implementation of the intervention and the trial design (intervention (IG) vs control group (CG)). Here, the focus is on the feasibility and exploratory results of the primary (combined endpoint of hospitalisation, falls and mortality) and secondary outcome measures (improving GP practices’ and patients’ knowledge of health-related resource use (FIMA), as well as improving patients’ Health Literacy (HLQ-G) and Satisfaction with Medication Information (SIMS-D)). Data were analysed according to the intention to treat principle.

**Results:**

Twelve GP practices were randomised (6 per IG/CG). Of 159 patients invited, 93 were included in the analyses (52 IG/41 CG). At t0 and t1, only few self-reported data from patients (5 resp. 10) or from the GP questionnaire (resp. 0) were missing. At least one survey perspective was available for each patient at both survey times. Overall, there were few missing items in the questionnaires, so the scores could not be calculated in 4-18% of cases (primary combined endpoint 9%), and there were no single items with increased missing values (*n* = 0 to 7). The concordance of the hospitalisation data from patients and GP practices was about 80%. Exploratory analyses showed no effects of the intervention on primary or secondary outcome measures.

**Conclusions:**

The primary combined endpoint was feasible. The secondary outcomes and survey methods used also proved feasible for GP practices and older multimorbid patients, with low missing rates. However, there was no hint of the influence of the intervention in the study groups in the explorative analyses.

**Trial registration:**

The trial was registered in the DRKS German Clinical Trials Register: registration number DRKS00027649 (date: 19.01.2022); http://www.drks.de/DRKS00027649.

**Supplementary Information:**

The online version contains supplementary material available at 10.1186/s12875-025-02774-5.

## Background

Several papers have emphasised the importance of continuity of care, which is a complex construct [1–3]. The aspect of ‘informational continuity’ [1] or more broadly ‘distributed work’ [3] is still insufficiently addressed at the interface of outpatient and inpatient care in Germany and beyond [4–8]. Additionally, there is neither sufficient information exchange between healthcare professionals nor between professionals and patients [7]. Lack of information, whether concerning medication or medical history [4, 9, 10], increases the risk of medication discontinuation [5, 11, 12] and adverse drug events, many of which would otherwise be preventable [13–15].

Older patients, in particular, are more frequently affected by polypharmacy [4, 16, 17]. Patients with multimorbidity and polypharmacy often experience medication changes [18, 19], such as discontinuation and restarts [20], as well as inappropriate prescribing [21, 22]. Medication changes pose a challenge to medication management – the complexity is increased by the mix of new medications, side effects and disease symptoms that are difficult to distinguish for patients [18]. Adherence can be poor due to communication barriers, such as a lack of both information and support for day-to-day medication management [18]. Sell and Schaefer (2020) showed that gaps in patients’ medication knowledge, which increase with the age of the patients, are a risk factor for drug-related problems [23]. In Germany, health literacy (the ability to understand and use health-related information) is generally low, especially among older people and those with chronic diseases [24]. In particular, digital health literacy (a special focus on understanding and using digital health information) is low, again with older people having the highest risk [24].

Indeed, also the majority of patient contacts in general practitioner (GP) practices are with multimorbid patients [25, 26], which require an effective management of their complex needs within the healthcare system. In many countries, the GP has a gatekeeper role requiring broad expertise and coordination of care between different healthcare professionals [26, 27]. Therefore, it is important for the GP to have access to all relevant information. This role is also the aim in Germany [27, 28], but there is a free choice of doctors and often only a limited overview on the part of health professionals of the medical services used [27]. Salm et al. (2023) have recently shown that the coordination of care is insufficient in older multimorbid patients in Germany [8]. Barriers to the exchange of medical information include strict data protection regulations [27] and the insufficient implementation of digital solutions in Germany, such as electronic patient records (ePA in Germany) [29]. Furthermore, inpatient and outpatient care are strictly separated in terms of organization and finances, making intersectoral care particularly challenging [30] including the prescription of medications. Internationally, patients have access to their visit notes online [31], with participation increasing over the years [32, 33]. In the US, a project called ‘open notes’ was launched in 2010 [34], which is characterised by mandatory easy online access since 2021 [35]. In contrast, in Germany, patients can inspect their medical documentation, but the barriers are higher. For instance, inspection has to be actively requested, and patients have to pay for copies [36]. The introduction of the ePA in Germany started as a pilot project in model regions in January 2025 and is intended to gradually become fully available with complete technical functionality in the future, with patients having the option to object and to decide which content is stored [37].

The pilot trial, HYPERION-TransCare (‘Heading to ContinuitY of Prescribing in EldeRly with MultImOrbidity iN Transitional Care’), tested the feasibility of the trial design and implementability of a new complex intervention aiming to improve the continuity of information at the interface between care providers but also the availability of information to patients. This should lead to a sustainable reduction in negative health-related outcomes in patients and empower patients’ health literacy.

The new complex intervention was developed in a participatory way with stakeholders from the outpatient and inpatient settings, including patients and informal caregivers in the previous first part of the study [38–40].

This paper presents the feasibility and first results of selected outcome measures of a cluster-randomised controlled pilot trial (cRCT) in older patients in primary care. The following issues are addressed:

- ▪ Feasibility of primary and selected secondary outcome measures
- ▪ Descriptive statistics of outcome measures and exploratory statistical analyses

## Methods

### Study design

A multi-centre cRCT was conducted. The entire trial period lasted from July 2021 to September 2024 (conception to analysis completion). The trial was accompanied by an explorative mixed-methods process evaluation which also addresses the feasibility of the selected study components within the trial setting. The study protocol [41] and results of the medication analyses [42], and the explorative process evaluation, as well as the recruitment experience will be published elsewhere.

The trial was funded by the Federal Ministry of Education and Research in Germany (BMBF; grant number (01GK1906A/01GK1906B)) and is registered in the German Clinical Trials Register (DRKS ID DRKS00027649). The trial complies with the Declaration of Helsinki and was approved by the responsible ethics committees. The presentation of the paper complies with the CONSORT 2010 checklist (see additional file 1). We used DeepL [43] and DeepL Write [44] to support the linguistic creation of the manuscript and to improve readability. After using these tools, the authors reviewed and edited the content as needed and take full responsibility for the content of the published article.

### Setting and participants

The trial was planned with the participation of ten GP practices from Hesse and ten GP practices from Saxony, each with ten multimorbid patients ≥ 65 years. GP practices were mainly recruited through the SaxoForN practice-based research network (PBRN), which has been established in the regions of Hesse and Saxony in Germany since 2020 (Forschungspraxennetz Allgemeinmedizin Dresden/Frankfurt am Main; [45–47]). SaxoForN enables us to represent a western and an eastern region of Germany, with both regions featuring rural and urban areas. Inclusion criteria for GP practices were as follows: all family doctors (specialists in general or internal medicine) that did not participate in the previous qualitative study (first part of this study) which aimed to co-develop the intervention [38]. Each GP practice in this trial consisted of a team of a GP and a healthcare assistant (HCA), who were free to divide the study tasks among themselves. Patients were recruited by the GP practices. Inclusion criteria for patients were as follows: age ≥ 65 years, ≥ 2 chronic diseases, ≥ 5 chronic medications, ≥ 1 hospitalisation in the last 12 months, and sufficient knowledge of German (reading and language comprehension). Patient exclusion criteria were: inability to give consent, and living in a nursing home or having participated in the previous qualitative study on the development of this intervention. Patients were also excluded if they had a severe mental disorder (ICD-10 F diagnoses) that, in the judgement of the GP, might interfere with their participation in the study [38].

**Fig. 1:**
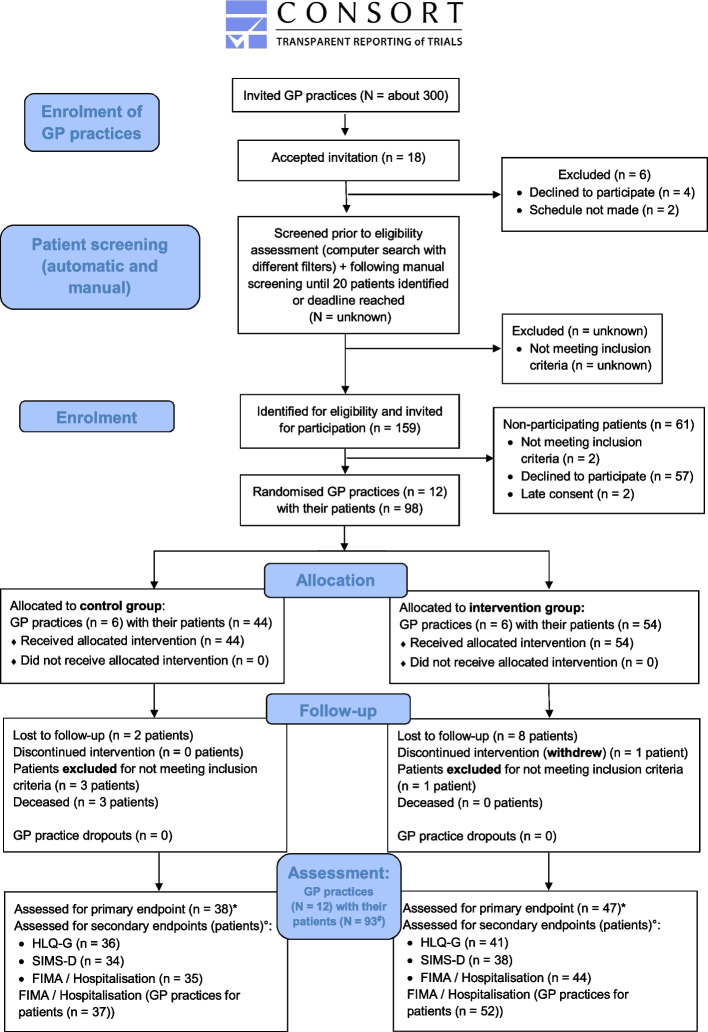
Flow diagram of the participants in the HYPERION-TransCare cRCT pilot trial

Participation by patients and GP practices was voluntary, based on signed informed consent and included financial compensation. An overview of the study participants (GP practices and patients) can be found in the CONSORT flow diagram [48] in Fig. 1.

### Study arms and intervention

This trial is a two-arm pilot trial with a control group (CG; care-as-usual) and an intervention group (IG) conducted under everyday conditions. Randomisation to the trial arms was carried out at the level of GP practice clusters after patient recruitment and the baseline survey (t0) by an independent member of the institute. All participants were blinded. Trained study staff ensured concealment of group allocation during interactions. In addition, the CG received a pseudo-intervention consisting of GP practice guidelines and a patient flyer (see next section).

Over six-months, the IG received a complex intervention including: a) a paper-based 'patient portfolio' (additional files 2 and 3), b) an accompanying 'GP practice checklist' (digital or paper-based) (additional file 4), and c) the regular support of GP practices to their patients in managing their patient portfolio. Patients recorded relevant health information, including diseases, treatments, and medications, with support from their GP practice. GP practices received and updated the patient medical information in the portfolio, using the checklist to manage updates.

Patients in the CG received care as usual, i.e. in accordance with German GP practice guidelines regarding management of polypharmacy and multimorbidity [49, 50]. The Multimorbidity Guideline recommends a holistic approach to patient care, considering not only known diseases and psychosocial factors but also individual preferences (shared decision-making) during consultations [50]. The Polypharmacy Guideline complements this with a structured process for the regular review, adjustment, and safe prescription of medications, ensuring patient involvement and education [49]. GP practices from both the IG and CG received the short version of these guidelines. In addition, all patients received a flyer, developed by the study staff, on how to share information with healthcare providers.

All GP practices received online training to conduct the study including recruiting strategies, materials, timelines, data collection and, if applicable, the intervention.

Throughout the study, the study team maintained regular contact with the participants (patients and GP practices) via e-mail or telephone to answer questions and provide support if needed. Method and design of the trial are described in detail in the published study protocol [41], to which no important changes were made during this trial.

### Pilot study outcome measures

The primary outcome is the combined endpoint composed of the information on:

- hospitalisation (item for both patients and GP practices in FIMA questionnaire (Fragebogen zur Inanspruchnahme medizinischer und nicht-medizinischer Versorgungsleistungen im Alter/questionnaire for the use of medical and non-medical services in old age) [51, 52]; at the beginning of the study (t0; baseline): ‘Have you/Has your patient been in hospital for inpatient care during the last 12 months?‘ / or at the end of the study (t1; after six months; follow-up):’…. during the past six months?’ – answer option: ‘Yes/No’)
- falls (self-developed questions for patients based on the World Health Organization (WHO) definition [53]: ‘In the last three months, have you involuntarily slipped, tripped, fallen or tumbled in such a way that you landed on the floor or a lower level?’ – answer option: ‘Yes/No’; ‘How many times have you fallen or crashed in the last three months?’ – free text answer); collected at t0 and t1
- mortality (question for GP practices: indication of date of death of the patient), collected at t1

Secondary outcomes were derived from standardised and validated questionnaires:

- patients’ satisfaction with medication-related information: The Satisfaction with Information about Medicines Scale, German version (SIMS-D) [54] consists of a total score for the Satisfaction with Information about Medicines (17 items) and the two subscales ‘Information on action and usage of medication’ (nine items) and ‘Information on potential problems of medication’ (eight items).
- patients’ health literacy: Health Literacy Questionnaire – German (HLQ-G) [55] consisting of nine health literacy domains with a total of 44 items: ‘Feeling understood and supported by healthcare providers‘, ‘Having sufficient information to manage my health‘, ‘Actively managing my health‘, ‘Social support for health‘, ‘Appraisal of health information‘, ‘Ability to actively engage with healthcare providers‘, ‘Navigating the healthcare system’, ‘Ability to find good health information’, and ‘Understanding health information well enough to know what to do’.
- medication and treatment-related information from patients‘ perspective: FIMA [51, 52] measures health-related resource use (including medication) in older people in seven categories [52]. The questionnaire consists of 14 items referring to a period of seven days, three months or 12 months, some of which have several sub-items, as well as socio-demographic and questionnaire-related items [51]. At t1, four items (on health insurance (how and where) and on educational and professional qualifications) were omitted for patients at t1 in order to save resources.
- medication and treatment-related information from GP practices‘ perspective: adapted FIMA. We slightly adapted the FIMA questionnaire for use in GP practices in agreement with the questionnaire authors at both measurement points to capture knowledge about patients' healthcare use from the GP practices’ perspective (e.g. instead of ‘Have you…?‘, ‘Has your patient… ?‘ was used), and reduced item detail: For example, the answer option ‘unknown‘ was added to all questions except socio-demographic questions, and further items on frequency or duration were largely deleted. Items about patients' educational and professional qualifications and the difficulty of completing the questionnaire were also removed. The item on ‘help in completing the questionnaire‘ was changed to ‘Who completed the questionnaire?‘. Items about health insurance (how and where), year of birth and sex of the patients were not asked at t1 in the GP practices in order to save resources.
  At t1, all items referring to a 12-month period in the FIMA were changed to a six-month period for both the patients’ and GP’ perspective, as this corresponds to the intervention period. In this study, we used the FIMA to assess the availability of health-related information by comparing the information provided by patients and the GP practices at t0 and t1.
- symptoms and/or side effects of the medication: selection of the NATIONAL CANCER INSTITUTE Patient Reported Outcomes version of the Common Terminology Criteria for Adverse Events – Items-German (NCI PRO-CTCAE® Items-GERMAN) [56, 57], consisting of seven items about symptoms in the last seven days, asked on a 5-point frequency or severity scale or with yes/no answer. There was also the option of reporting other symptoms. The analysis and presentation of this outcome is not the focus of this paper.

In addition to the primary and secondary outcomes, the following self-developed questions (structure and social data) were asked patients only at t0:

- living arrangement (living alone; with others; assisted living/retirement home/nursing home)
- medication plan (sources and updates)
- ePA (familiarity and use)

The GP practices were also asked at t0 to rate the illnesses of their study patients in 14 disease groups (based on the Cumulative Illness Rating Scale (CIRS) [58]) and to provide their own socio-demographic characteristics (structure and social data) including age, sex, practice size, etc. (self-developed questionnaire).

### Data collection procedures

**Fig. 2:**
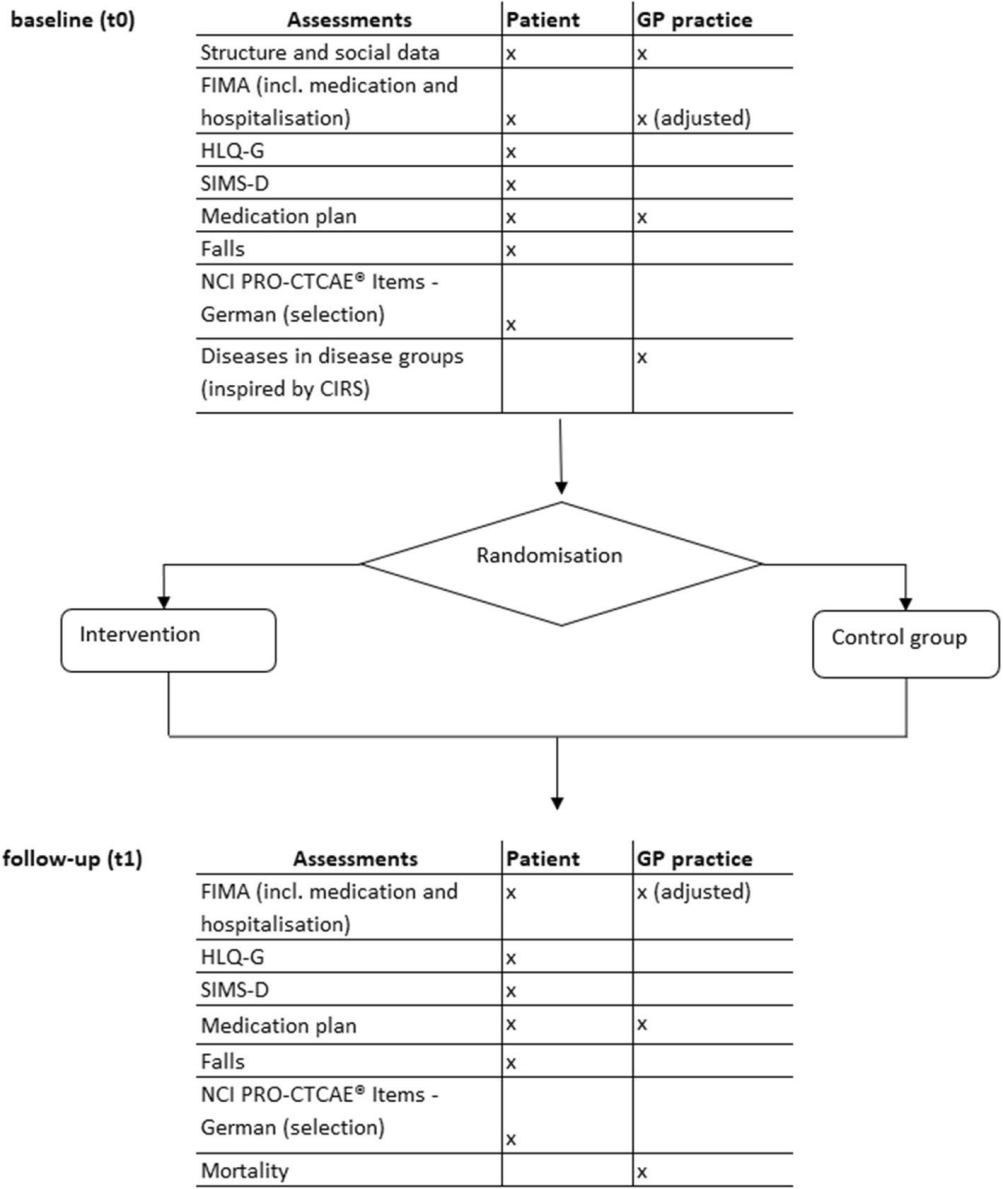
Time schedule and assessments for participants in the HYPERION-TransCare trial. The assessments used (shown in this figure with their abbreviations) are explained in detail in the main text

An overview of the time schedule with questionnaires is given in Fig. 2.

#### patients

Patients received a pseudonymised paper-based questionnaire with a postage paid return envelope at t0 and t1.

#### GP practices

The surveys in the GP practices were conducted digitally via an electronic data capture tool (Research Electronic Data Capture (REDCap) [59]) hosted at the Dresden University of Technology. At t0 and t1, the GP practices were asked to document the primary and secondary outcomes of their participating patients in a pseudonymised form.

#### Analysis

This paper focuses on the feasibility of the collection of selected outcome measures. The analyses were performed by the collaborating partner, Ruhr University Bochum, using the statistical software R, version 4.1.0. Missing and descriptive analyses were performed and possible effects of the intervention were explored. Some data, such as free text entries, medication data, disease group data, and comparison of physician characteristics with national data, were categorised (where applicable) and analysed descriptively by study staff using SPSS version 29 and Excel. All analyses followed the intention-to-treat principle.

### Calculation of the primary combined endpoint

The combined endpoint was assessed dichotomously as follows (0 = does not apply, 1 = applies): If at least one of the three aspects (hospitalisation, falls, mortality) was fulfilled, then the value 1 was assigned. The score was only set as missing if both hospitalisation and falls were missing for living patients, or if one was missing and the other one was not applicable. There was no missing information on mortality because the GP practices recorded deaths and there was regular contact with the study team for those patients who were not recorded as deceased, so this information was available on all patients by the end of the trial.

### Calculation of the secondary outcomes

For missing analyses of secondary outcomes, only patient cases for which a questionnaire was received at t0 or t1 respectively were considered. A (total) score or subscore/domain was not calculated if at least one item was missing; the patient case was excluded from that specific analysis. As this is a pilot trial, missing values were not imputed.

The analysis of SIMS-D was dichotomous according to the validation publication of the German version of the questionnaire. Items were scored on a 5-point rating scale (too much – too little – none received—none needed – about right), with the first three mentioned response categories coded as 0 and the last two coded as 1. A sum score was calculated for each subscale (maximum value of 8 or 9) and a total sum score of all items (maximum value of 17), with higher scores reflecting higher levels of satisfaction. [54]

For HLQ-G, the items of the first five domains were asked on a 4-point Likert scale (from 1 = strongly disagree to 4 = strongly agree) and the items of the last four domains were asked on a 5-point Likert scale (from 1 = cannot do to 5 = very easy) [55, 60]. For the analysis, the values of the individual items per scale were summed and divided by the number of items [60].

For FIMA, no score is provided for analysis [51]. The presentation of the results is descriptive, separated into the two study groups and includes a comparison of practice and patient data (agreement and disagreement regarding ‘hospitalisation’; see above ‘Analysis’).

### Calculation of educational level

The patients' education was surveyed in two variables with categories (educational and professional qualification) of the German education system, and then classified (1 = low; 2 = medium and 3 = high level of education). The educational level was derived from the highest achieved category of the two variables. This classification is based on the Comparative Analysis of Social Mobility in Industrial Nations (CASMIN) educational classification [61].

The effect on the combined endpoint was analysed using a mixed logistic regression model with study group, age and sex as fixed effects and cluster added as a random effect. A similar logistic mixed model with study group and age as fixed effects (but without sex due to fitting problems) and cluster as a random effect was used for secondary binary endpoints, e.g. hospitalisation, and falls. For hospitalisation, an additional separate analysis was performed using a mixed negative binomial model. For all scores (HLQ and SIMS) linear mixed effect models were used with study group, the baseline value of the score and sex as fixed effects and cluster as a random effect. The significance level used was α = 5%. Furthermore, agreement and disagreement in FIMA responses between patients and GPs are presented as an example for the reported number of hospitalisations, as hospitalisation is part of the primary outcome and therefore particularly important.

## Results

The trial was conducted from March to December 2022. Fourteen GP practices with a total of 100 patients formally agreed to participate in the study. Two patients provided consent after the start of the study, while one patient withdrew during the study. In addition, two GP practices were excluded because they started the study too late (no patients had been enrolled). Four more patients were excluded because they did not meet the inclusion or met the exclusion criteria (they had not been hospitalised or had fewer than five medications). This resulted in 12 GP practices with 93 patients who participated in the study and whose data were included in the analyses (see Fig. 1).

### GP practice characteristics

The participating GP practices were located in the two federal states of Hesse (*n* = 8) and Saxony (*n* = 4) in both rural and urban areas. Of the 12 participating GPs, four were female (33.3%) and the average age of the physicians was 52.8 years (SD = 9.5). Most were practice owners (91.7%; *n* = 11) and only one was an employed physician. They worked in a single practice (50.0%; *n* = 6) or in a joint practice (50.0%; *n* = 6). Most GPs reported previous research experience (*n* = 10; 83.3%). All 12 participating HCAs were female (100%) and had a mean age of 40.5 years (SD = 13.0). Physician characteristics were compared descriptively with nationwide data from the Federal Register of Physicians of the National Association of Statutory Health Insurance Physicians (Kassenärztliche Bundesvereinigung (KBV); NASHIP) in Germany [62]. The very small sample showed that, compared with the national distribution, study participants were slightly more likely to be male, to come from similar types of practice (except healthcare centres), and to be slightly younger (see table in additional file 5).

### Characteristics of GP practices’ study documentation

Of the 93 patients included in the analyses, GP practices documented data for 87 patients at t0 and for all 90 surviving patients at t1. Missing data from GP practices at t0 were all from one GP practice that failed to document within the designated timeframe due to time constraints. GP practices took approximately 23 min (SD = 16.8) to complete the questionnaire per patient at t0 and approximately 18 min (SD = 15.9; shortened questionnaire) at t1. The final open-ended free text question (opportunity for feedback) of the questionnaire was used by the GP practices for 22 patients at t1. The majority of responses related to difficulties with the questionnaire (63.6%; *n* = 14), but there were also several identical entries from the same practice. In addition, there were occasional comments, e.g. on the design and construction of the questionnaire or general statements (rated as ‘not applicable’). The questionnaires were completed by either the GP, the HCA or both together.

### Patient characteristics

A total of 52 patients (26 female) were included in the IG and 41 patients (11 female) in the CG. Patient characteristics are presented below based on patient-reported data at t0. However, five of the 93 patients did not return a questionnaire (see next section Characteristics of patients’ study documentation).

The following section, as well as Table 1 below, refers to the 88 questionnaires returned by patients. The mean age of the participating patients was 77.9 years (SD = 7.0); 34 (39.5%) were female. The educational level of the majority was medium (67.1%; *n* = 57), followed by high (28.2%; *n* = 24) and low (4.7%; *n* = 4). All patient characteristics reported by patients at t0 are shown by study group in Table 1. Apart from sex (more men in the CG), there are no obvious differences between the two groups. Table 2 gives an overview of the morbidity of the patients at t0. Overall, fewer disease groups were reported for patients in the CG.

**Table 1:** Patient characteristics reported by patients at t0

	IG (*n* = 49)	CG (*n* = 39)	Total (*n* = 88)
age			
n-miss	2	0	2
mean (SD)	79.1 (7.0)	76.4 (6.8)	77.9 (7.0)
range	65.0 - 94.0	66.0 - 89.0	65.0 - 94.0
sex			
n-miss	2	0	2
female (%)	24 (51.1)	10 (25.6)	34 (39.5)
male (%)	23 (48.9)	29 (74.4)	52 (60.5)
diverse (%)	0 (0.0)	0 (0.0)	0 (0.0)
marital status			
n-miss	2	1	3
married (%)	27 (57.4)	28 (73.7)	55 (64.7)
single (%)	4 (8.5)	0 (0.0)	4 (4.7)
divorced (%)	2 (4.3)	4 (10.5)	6 (7.1)
widowed (%)	14 (29.8)	6 (15.8)	20 (23.5)
living arrangement			
n-miss	2	1	3
living alone (%)	17 (36.2)	6 (15.8)	23 (27.1)
with other persons (partner/family) (%)	24 (51.1)	32 (84.2)	56 (65.9)
assisted living or retirement/nursing home (%)	6 (12.8)	0 (0.0)	6 (7.1)
educational level			
n-miss	2	1	3
low level of education (%)	2 (4.3)	2 (5.3)	4 (4.7)
medium level of education (%)	28 (59.6)	29 (76.3)	57 (67.1)
high level of education (%)	17 (36.2)	7 (18.4)	24 (28.2)
different drugs taken in the last 7 days			
n-miss	2	0	2
median (interquartile range)	7.0 (5.0 - 9.0)	8.0 (6.0 - 10.0)	7.5 (5.0 - 9.0)
level of care (German classification) n-miss	5	0	5
no level of care (%)	31 (70.5)	26 (66.7)	57 (68.7)
level 1 (%)	3 (6.8)	2 (5.1)	5 (6.0)
level 2 (%)	9 (20.5)	5 (12.8)	14 (16.9)
level 3 (%)	1 (2.3)	6 (15.4)	7 (8.4)
level 4 (%)	0 (0.0)	0 (0.0)	0 (0.0)
level 5 (%)	0 (0.0)	0 (0.0)	0 (0.0)
federal state			
n-miss	0	0	0
Hesse (%)	32 (65.3)	22 (56.4)	54 (61.4)
Saxony (%)	17 (34.7)	17 (43.6)	34 (38.6)

*n* number of participants, *IG* intervention group, *CG* control group, % percent

**Table 2:** Number of disease groups reported by GP practices about their patients at t0

	IG (*n* = 46)	CG (*n* = 41)	Total (*n* = 87)
number of disease groups			
2–4 disease groups (%)	8 (17.4)	13 (31.7)	21 (24.1)
5–7 disease groups (%)	16 (34.8)	20 (48.8)	36 (41.4)
8–10 disease groups (%)	20 (43.5)	6 (14.6)	26 (29.9)
11–13 disease groups (%)	2 (4.3)	2 (4.9)	4 (4.6)

*n* number of participants, *IG* intervention group, *CG* control group, % percent

### Characteristics of patients’ study documentation

Of the 93 patients whose data were included in the analysis, 88 patients self-reported data at t0 and 80 patients at t1, with eight missings in the IG. Three patients from the CG died during the trial. Of the five cases of missing data at t0, one was due to the data arriving at the institute after the end of the data collection period; the reasons in the other four cases remain unclear. Of the ten cases of missing data at t1 (excluding the three deaths), one was due to deterioration in health, which meant that the questionnaire could not be completed. The reasons for the other nine cases remain unclear. Only one patient did not complete the questionnaire at both t0 and t1. At least one survey perspective, patient and/or GP practice, was available at both t0 and t1 for all patients.

On average, patients took approximately 63 min (t0; SD = 39.0) or 58 min resp. (t1; SD = 32.3; shortened questionnaire) to complete the questionnaire. The majority completed the questionnaire at t1 independently, without help from others (78%; *n* = 61), and rated the completion as 'easy' (79,5%; *n* = 62). On the other hand, 5% (*n* = 4) said it was ‘impossible to complete without help’. Where patients did receive help to complete the questionnaire at t1, this was most often from relatives (88%; *n* = 15) or in the remaining cases from a carer (12%; *n* = 2). The final open-ended free text question (feedback opportunity) of the questionnaire was used by 18 patients at t1. Most respondents to it reported difficulties with the questionnaire (33%; *n* = 6), followed by general comments about the healthcare system (28%; *n* = 5), but there were also occasional comments e.g. about the design and construction of the questionnaire, the long length, the need for assistance in completing the questionnaire, or personal statements (rated as ‘not applicable’).

### Results: primary outcome, secondary outcomes and missings

The combined primary endpoint could not be calculated at t1 for eight patients (8.6%; 5 IG and 3 CG patients) due to missing data.

For a small majority (56.5%; *n* = 48), the combined endpoint was reached. There were no differences in the combined endpoint between the two study groups (see Table 3). Logistic regression analysis also showed no differences in the combined endpoint or its sub-aspects (hospitalisation, falls). For mortality, the model did not fit (only deceased in the CG). The additional analyses of hospitalisation in the negative binomial model also showed no effect of age or study group on the likelihood of participants being hospitalised.

**Table 3:** Result for the primary endpoint and its components, odds ratios (OR), 95%-confidence interval (CI) and p-values were calculated from mixed logistic regression model with study group and age as fixed effects and cluster added as a random effect

	IG (*n* = 52)	CG (*n* = 41)	OR CG vs IG	95%-CI	*p*-value
**combined endpoint (t1)**			0.95	0.33 - 2.75	> 0.9
n-miss	5	3			
applies (at least 1 of 3 aspects) (%)	27 (57.4%)	21 (55.3%)			
**hospitalisation (patient data)**			0.91	0.32 - 2.61	0.9
n-miss	8	6			
yes	12 (27.3%)	9 (25.7%)			
**hospitalisation (GP practice data)**			1.18	0.44 - 3.14	0.7
n-miss	0	4			
yes	15 (28.8%)	12 (32.4%)			
**falls**			0.77	0.25 - 2.35	0.6
n-miss	9	5			
yes	13 (30.2%)	7 (19.4%)			

*n* number of participants, *IG* intervention group, *CG* control group, % percent, *OR* odds ratio, *CI* confidence interval

### Secondary outcomes

Tables 4 to 5 provide an overview of the SIMS-D results. On the 88 questionnaires received at t0, the SIMS-D subscale 1 could not be calculated for nine patients (10%), subscale 2 for 12 patients (14%) and total sum score for 16 patients (18%) due to missing data. Of the 80 questionnaires received at t1, subscale 1 could not be calculated for eight patients (10%), subscale 2 for ten patients (13%) and total sum score for 12 patients (15%). There was not a particularly high number of missing values for any item on the SIMS-D questionnaire (2–6 missing values per item), with three patients not completing subscale 1 at all, one of whom did not complete subscale 2 either. Also, for t1, three patients missed subscale 1 completely, two of whom also missed subscale 2 completely. The number of missing values was also not particularly high for any of the items (maximum 3–6 missing values per item). The group comparison showed lower missings in the CG in all three scales at t1; at t0 this applies only to subscale 1.

**Table 4:** SIMS-D results at t0 and t1

	t0			t1		
	IG (*n* = 49)	CG (*n* = 39)	Total (*n* = 88)	IG (*n* = 44)	CG (*n* = 36)	Total (*n* = 80)
**subscale 1**						
**information on action and usage of medication**						
n-miss	6	3	9	6	2	8
mean (SD)	7.5 (1.8)	6.6 (2.7)	7.1 (2.3)	6.2 (2.4)	6.6 (2.4)	6.4 (2.4)
range	1.0 - 9.0	1.0 - 9.0	1.0 - 9.0	0.0 - 9.0	2.0 - 9.0	0.0 - 9.0
**subscale 2**						
**information on potential problems of medication**						
n-miss	6	6	12	8	2	10
mean (SD)	4.0 (2.8)	4.5 (3.1)	4.3 (2.9)	3.5 (3.1)	4.8 (2.8)	4.1 (3.0)
range	0.0 - 8.0	0.0 - 8.0	0.0 - 8.0	0.0 - 8.0	0.0 - 8.0	0.0 - 8.0
**sum score SIMS-D**						
n-miss	9	7	16	8	4	12
mean (SD)	11.6 (3.7)	11.4 (5.3)	11.5 (4.5)	9.7 (5.1)	11.3 (4.7)	10.4 (4.9)
range	1.0 - 17.0	2.0 - 17.0	1.0 - 17.0	1.0 - 17.0	3.0 - 17.0	1.0 - 17.0

*n* number of participants, *IG* intervention group, *CG* control group, *SD* standard deviation, % percent

**Table 5:** Mixed linear regression of SIMS-D with random cluster effect and baseline score and sex as covariates

Group	Characteristic	Beta	95% CI^1^	p-value
sum score SIMS-D	CG vs IG	1.3	−1.1, 3.6	0.3
subscale 1: information on action and usage of medication	CG vs IG	0.6	−0.6, 1.8	0.3
subscale 2: information on potential problems of medication	CG vs IG	0.5	−1.4, 2.4	0.5

^1^CI = confidence interval

The SIMS-D showed similar scores on both subscales and the sum score at both survey times in both study groups (see Table 4). There were also no significant effects in the mixed linear regression (see Table 5).

Tables 6 to 7 provide an overview of the HLQ-G results. Of the 88 questionnaires received at t0, between 76 and 84 complete patient cases (86–95%) could be included in the calculation of the nine HLQ-G subscales. At t1 there were 72–77 complete cases from the 80 questionnaires (90–96%) that were included in analysis. There was not a particularly high number of missing values for any item: 1–7 missing values per item at t0; 0–6 missing values per item at t1. The group comparison showed higher missings in the CG for the subscales HLQ6 to HLQ9 at t0. In contrast, at t1 the missing values were quite similar in both groups (HLQ5 to HLQ9), but for the scales HLQ1 to HLQ4 there were no missing values at all in the CG.

**Table 6:** HLQ-G results at t0 and t1

	t0			t1		
	IG (*n* = 49)	CG (*n* = 39)	Total (*n* = 88)	IG (*n* = 44)	CG (*n* = 36)	Total (*n* = 80)
HLQ1:						
Feeling understood and supported by healthcare providers						
n-miss	3	3	6	3	0	3
mean (SD)	3.3 (0.5)	3.2 (0.4)	3.3 (0.4)	3.2 (0.5)	3.3 (0.5)	3.2 (0.5)
range	2.5 - 4.0	2.5 - 4.0	2.5 - 4.0	1.8 - 4.0	2.3 - 4.0	1.8 - 4.0
HLQ2:						
Having sufficient information to manage my health						
n-miss	4	3	7	5	0	5
mean (SD)	3.0 (0.5)	3.0 (0.4)	3.0 (0.4)	2.9 (0.4)	3.0 (0.4)	2.9 (0.4)
range	2.0 - 4.0	2.0 - 4.0	2.0 - 4.0	1.8 - 3.8	2.0 - 4.0	1.8 - 4.0
HLQ3:						
Actively managing my health						
n-miss	4	2	6	5	0	5
mean (SD)	2.9 (0.5)	2.8 (0.4)	2.9 (0.4)	2.8 (0.4)	2.7 (0.4)	2.8 (0.4)
range	1.8 - 4.0	2.0 - 3.8	1.8 - 4.0	2.0 - 4.0	1.6 - 4.0	1.6 - 4.0
HLQ4:						
Social support for health						
n-miss	2	2	4	3	0	3
mean (SD)	3.0 (0.6)	3.0 (0.4)	3.0 (0.5)	3.0 (0.6)	3.0 (0.4)	3.0 (0.5)
range	1.8 - 4.0	2.0 - 4.0	1.8 - 4.0	1.8 - 4.0	2.0 - 4.0	1.8 - 4.0
HLQ5:						
Appraisal of health information						
n-miss	2	2	4	4	2	6
mean (SD)	2.5 (0.6)	2.4 (0.4)	2.5 (0.5)	2.5 (0.6)	2.5 (0.5)	2.5 (0.5)
range	1.2 - 4.0	1.6 - 3.4	1.2 - 4.0	1.2 - 3.6	1.8 - 4.0	1.2 - 4.0
HLQ6:						
Ability to actively engage with healthcare providers						
n-miss	1	5	6	3	2	5
mean (SD)	3.7 (0.7)	3.6 (0.6)	3.7 (0.7)	3.4 (0.8)	3.8 (0.6)	3.6 (0.7)
range	1.8 - 5.0	2.4 - 4.8	1.8 - 5.0	2.0 - 5.0	2.8 - 5.0	2.0 - 5.0
HLQ7:						
Navigating the healthcare system						
n-miss	4	7	11	5	3	8
mean (SD)	3.6 (0.6)	3.4 (0.6)	3.5 (0.6)	3.3 (0.8)	3.4 (0.7)	3.3 (0.8)
range	2.3 - 4.5	2.2 - 4.3	2.2 - 4.5	1.7 - 4.8	2.0 - 4.8	1.67 - 4.8
HLQ8:						
Ability to find good health information						
n-miss	4	8	12	3	2	5
mean (SD)	3.5 (0.7)	3.3 (0.6)	3.4 (0.6)	3.3 (0.7)	3.4 (0.7)	3.4 (0.7)
range	1.6 - 4.6	1.8 - 4.2	1.6 - 4.6	1.2 - 4.6	1.6 - 4.6	1.2 - 4.6
HLQ9:						
Understanding health information well enough to know what to do						
n-miss	2	5	7	3	1	4
mean (SD)	3.7 (0.8)	3.3 (0.7)	3.5 (0.7)	3.5 (0.8)	3.6 (0.7)	3.5 (0.7)
range	1.6 - 5.0	1.6 - 4.4	1.6 - 5.0	1.8 - 5.0	1.6 - 4.6	1.6 - 5.0

*n* number of participants, *IG* intervention group, *CG* control group, *SD* standard deviation, % percent

**Table 7:** Mixed linear regression of HLQ-G with random cluster effect and baseline score and sex as covariate

Group	Characteristic	Beta	95% CI^1^	p-value
HLQ1: Feeling understood and supported by healthcare providers	CG vs IG	0.17	−0.03, 0.37	0.09
HLQ2: Having sufficient information to manage my health	CG vs IG	0.11	−0.08, 0.30	0.2
HLQ3: Actively managing my health	CG vs IG	−0.07	−0.29, 0.14	0.5
HLQ4: Social support for health	CG vs IG	−0.01	−0.20, 0.18	0.9
HLQ5: Appraisal of health information	CG vs IG	−0.01	−0.36, 0.34	> 0.9
HLQ6: Ability to actively engage with healthcare providers	CG vs IG	0.40	0.16, 0.65	0.002
HLQ7: Navigating the healthcare system	CG vs IG	0.23	−0.16, 0.62	0.2
HLQ8: Ability to find good health information	CG vs IG	0.26	0.00, 0.52	0.054
HLQ9: Understanding health information well enough to know what to do	CG vs IG	0.29	−0.08, 0.66	0.11

^1^CI = confidence interval

The HLQ-G showed similar scores on all nine subscales at both survey times in both study groups (see Table 6). Apart from the subscale HLQ6, there were also no significant effects in the mixed linear regression (see Table 7).

Tables 8 to 10 provide an overview of the FIMA results for ‘hospitalisation’. The data were derived from two sources: patient self-reported and information from GP practices about patients. There were only few (0–1) missings in the returned questionnaires for this item in these two groups and at both survey times. Most patients and GP practices reported hospitalisation at t0 (inclusion criterion for patients; see Tables 8 and 9). At t1, most patients and GP practices reported no hospitalisation, with no differences between the two study groups.

**Table 8:** Hospitalisation in FIMA at **t0** and **t1** as self-reported by patients

		t0		t1		
	IG (*n* = 49)	CG (*n* = 39)	Total (*n* = 88)	IG (*n* = 44)	CG (*n* = 36)	Total (*n* = 80)
**Hospitalisation**						
n-miss	0	1	1	0	1	1
no (%)	9 (18.4)	4 (10.5)	13 (14.9)	32 (72.7)	26 (74.3)	58 (73.4)
yes (%)	40 (81.6)	34 (89.5)	74 (85.1)	12 (27.3)	9 (25.7)	21 (26.6)

*n* number of participants, *IG* intervention group, *CG* control group, % percent

**Table 9:** Hospitalisation in FIMA at **t0** and **t1** as reported by GP practices about their patients

	t0			t1		
	IG (*n* = 46)	CG (*n* = 41)	Total (*n* = 87)	IG (*n* = 52)	CG (*n* = 38)	Total (*n* = 90)
**Hospitalisation**						
n-miss	0	0	0	0	1	1
no (%)	2 (4.3)	1 (2.4)	3 (3.4)	37 (71.2)	25 (67.6)	62 (69.7)
yes (%)	44 (95.7)	40 (97.6)	84 (96.6)	15 (28.8)	12 (32.4)	27 (30.3)
unknown (%)	0 (0.0)	0 (0.0)	0 (0.0)	0 (0.0)	0 (0.0)	0 (0.0)

*n* number of participants, *IG* intervention group, *CG* control group, % percent

The additional 'unknown' option added to the item for GP practices was never used. Table 10 shows a comparison of the information on hospitalisation provided by patients and GP practices at both survey points. If the missing data are excluded from the total, the agreement in the IG is about 80% (t0 = 79.1%; t1 = 81.9%) and about 85% in the CG (t0 = 86.8%; t1 = 82.4%).

**Table 10:** Comparison of data from patients and practices about their patients as absolute numbers for ‘hospitalisation’

		Self-reports by patients															
	**Hospitalisation**	t0: IG				t1: IG				t0: CG				t1: CG			
		No	Yes	Missing	**Total**	No	Yes	Missing	**Total**	No	Yes	Missing	**Total**	No	Yes	Missing	**Total**
Reports by GP practices	no	1	1	0	2	28	4	5	37	0	1	0	1	21	2	2	25
	yes	8	33	3	44	4	8	3	15	4	33	3	40	4	7	1	12
	unknown	0	0	0	0	0	0	0	0	0	0	0	0	0	0	0	0
	missing	0	6	0	6	0	0	0	0	0	0	0	0	1	0	3	4
	**Total**	9	40	3	52	32	12	8	52	4	34	3	41	26	9	6	41

*IG* intervention group, *CG* control group

## Discussion

The trial proved the methodological feasibility of the outcome measures used. The combined endpoint of hospitalisation, falls and mortality was methodologically feasible. There were only few missing data. The analysis also considered the individual components of the combined endpoint separately, as endpoints of different clinical relevance and frequency were combined in the combined endpoint (hospitalisation, falls and mortality) [63, 64].

The HLQ-G, SIMS-D and the FIMA item on hospitalisation all showed low missing values and no issues with individual items. In a larger main study, the missing values could be imputed appropriately with higher case numbers. In other studies using the HLQ-G, cases with missing values were excluded completely [65], or the values were replaced if less than half of the items were missing [55, 66, 67], or the scale score was classified as missing if half of the items were missing [68]. As previously stated, the present trial is a feasibility study, so no imputation was performed. Therefore, the observed missing data per item was higher in the present study (maximum 8%) than in the German validation study (maximum 5.5%; average 2.6%) [55] or in the international application [69–71]. The SIMS-D missing values in the present trial (18% and 15%) are similar to those in two German studies of chronically ill adults. In these studies, only complete SIMS-D questionnaires were used and incomplete ones were excluded, which affected about 20% of the returned questionnaires [54, 72]. In contrast, the study by Klewitz et al. (2019) had a lower miss rate (10%), however the patients were younger on average (median age: 51 years) [73]. This is consistent with the finding that, on average, older patients were more likely to have missing items in SIMS-D than younger patients (mean = 72.7 years vs mean = 68.3 years) [54]. Reports from patients or GP practices in the FIMA about patients who had not been hospitalised were clarified individually with the GP practices, as this was one of the inclusion criteria. It turned out that there was a considerable time lag between patient identification and completion of the questionnaire. This meant that in some cases hospitalisation was now slightly longer than 12 months ago. In the future, this item should be asked in quarters or by specifying the exact time frame for checking the inclusion criteria.

Overall, participants seemed to cope well with the items, as indicated by the low missing rates and the general feedback on the open-ended free text question. Conversely, it was also confirmed that the explicit reference to support was important for the patient collective, as some individuals found it difficult or impossible to complete the questionnaire without help. The low numbers of missings and low drop-out rates were achieved despite the high burden of the trial, which was reflected in the long time needed to complete the questionnaires, which was potentially stressful for older patients as well as for GP practices in their busy daily schedules. Average completion times were slightly shorter at t1 (GP practice from 23 to 18 min; patients from 63 to 58 min), which may be due to the slightly shortened FIMA questionnaire used at t1 in order to save resources, but also an effect of repeating the questionnaire.

The low drop-out and missing rates also show that the patients and GP practices who gave their consent were reliable participants. This may be because of the fact that all the GP practices were research practices in the SaxoForN PBRN. The resulting closer ties to the study team's institute and possibly greater research experience and affinity may have contributed to this. On the other hand, the study staff also maintained regular personal contact with the GP practices and patients during the trial. This was reflected in good availability to answer their questions and, conversely, in proactive contact during each step of the trial. In addition, the payment of the expense allowance after t0 may also have contributed to the desired effect of participants completing the study.

The exploratory analyses in this complex intervention pilot trial with GP practices and older multimorbid patients in Germany (Hesse and Saxony) failed to suggest that the intervention affected the primary or secondary outcomes. Only in the HLQ subscale 6 'ability to actively engage with healthcare providers' was a significant effect found in the regression analysis, with an improvement in the CG. The reasons for this remain unclear and could be coincidental given the small sample and the large number of tests. However, this pilot and feasibility trial was not designed to examine the efficacy of the intervention. A larger sample size would be needed to provide this evidence.

For the secondary outcomes, the present trial showed comparable scores on the HLQ-G scales to those observed in another German study of myocardial infarction patients [65]. The mean scores of the SIMS scales showed similar orders of magnitude, with dissatisfaction also tending to be reported regarding 'information on potential problems of medication’ [54, 72]. Overall, this shows a consistent effect, which has also been found in other studies conducted not only in Germany [73–75] but also internationally [76, 77], and was confirmed in a scoping review: ‘[…], patients most frequently requested information on adverse drug reactions (ADRs) and drug–drug interactions (DDIs)’ [78]. The FIMA is a commonly used instrument to estimate healthcare costs [79, 80] or to analyse referral trajectories [81]. To the best of our knowledge, this is the first trial to adapt the FIMA for completion by GP practices for their patients and to compare the information from practices and patients. The application proved to be feasible with a high agreement of information using hospitalisation data as an example. The additional 'unknown' option that was added to the item was never used. Accordingly, it does not seem to be necessary and could be omitted.

### Strengths and limitations

The findings of this feasibility study provide valuable insights for the adaptation of the subsequent main study. There were no protocol changes in this trial.

A recent study from Germany, which tested an intervention for nursing home residents to improve interprofessional collaboration in a cRCT, also failed to show an effect on hospital admissions and measures such as mortality and falls over a 12-month period [82]. The possible confounding factors, such as the underestimation of the intra-cluster correlation or the effect of the complex intervention on other important outcomes [82], must therefore also be considered in the main study.

Furthermore, it should be noted that the use of self-report instruments (patient-reported questionnaire data) can introduce sources of error. The large number of questions and the extended time required to complete them always carry the risk of response bias, especially among older, multimorbid patients, for whom this could also pose an additional burden. Nevertheless, it was reported that the majority of participating patients managed instrument completion well and described it as 'easy’. However, the time and effort required for the implementation of the intervention, both from patients and healthcare professionals, also need to be addressed and should be explored in a future study.

Regular contact with the study participants proved to be successful but also very time-consuming. However, it is questionable how realistic this would be with a larger number of participants and would need to be explicitly considered when designing any trial.

Nevertheless, the recruitment target was not met, neither in terms of the number of GP practices participating nor the number of patients. Frequent feedback from GP practices was a current time constraint, partly due to the ongoing COVID-19 pandemic and the associated regulations and high patient volumes in GP practices. In addition, GP practices may benefit from having more time to recruit patients. This extension could help to ensure that the study meets its recruitment targets, thereby enhancing the validity and reliability of the research findings. Further details regarding practice and patient recruitment will be described elsewhere.

The present findings suggest that the FIMA can be well used to survey practitioners and to compare the knowledge of different stakeholders (e.g. GP practices and patients). A validation study is needed to provide further evidence in this regard.

## Conclusion

This pilot trial provided important lessons for the design of a main study to analyse the effectiveness of the intervention. Key aspects include the recruitment from amongst a pool of research practices and close contact between the study team and participating practices and patients, which contributed to low missing data and dropout rates. However, a substantial time investment had to be taken into account by the study staff in the study planning, along with additional time for practice during recruitment. The outcomes used proved to be methodologically feasible despite the considerable effort required to complete the questionnaires in the setting of GP practices and older multimorbid patients. Additionally, FIMA shows potential not only as a self-report but also for comparing data across different stakeholders, though validation is needed. The findings may have implications for further interventional studies in primary care. In routine care, the findings will be important for the completion and usage of the patient-centered electronic health record, which was introduced starting January 2025 in Germany. However, the evaluation of effectiveness of the intervention and the permanent integration into everyday care transferability of the results needs to be addressed in a subsequent study phase.

## Supplementary Information

Additional file 1. CONSORT 2010 checklist.

Additional file 2. Table of contents of the patient portfolio.

Additional file 3. ‘Key patient data’ as part of the patient portfolio.

Additional file 4. Accompanying GP practice checklist.

Additional file 5. Comparison of HYPERION-TransCare physician characteristics with NASHIP data.

## Data Availability

Materials (in German language) and data (fully anonymised) generated in this study are available from the corresponding author upon reasonable request.

## Abbreviations

ADRs: Adverse drug reactions
BMBF: Federal Ministry of Education and Research in Germany
CG: Control group
CIRS: Cumulative Illness Rating Scale
cRCT: Cluster-randomised controlled trial
DDIs: Drug–drug interactions
DRKS: German Clinical Trials Register
ePA: German electronic patient records
FIMA: Fragebogen zur Inanspruchnahme medizinischer und nichtmedizinischer Versorgungsleistungen im Alter/questionnaire for the use of medical and non-medical services in old age
GP: General practitioner
HCA: Healthcare assistant (German job title, includes three years of professional training. HCAs perform a variety of tasks (organisational, supportive, link between doctors and patients).
HLQ-G: Health Literacy Questionnaire – German
Hyperion-TransCare: Heading to ContinuitY of Prescribing in EldeRly with MultImOrbidity iN Transitional Care
ICD 10: International Statistical Classification of Diseases and Related Health Problems - 10th revision
IG: Intervention group
NASHIP: National Association of Statutory Health Insurance Physicians (Kassenärztliche Bundesvereinigung; KBV)
N: Number base population
n: Number subgroup
NCI PRO-CTCAE® Items-GERMAN: NATIONAL CANCER INSTITUTE Patient-Reported Outcomes version of the Common Terminology Criteria for Adverse Events – Items - German
PBRN: practice-based research network
SaxoForN: Forschungspraxennetz Allgemeinmedizin Dresden/Frankfurt am Main
SIMS-D: Satisfaction with Information about Medicines Scale, German version
t0: survey at the beginning of the trial / baseline
t1: survey at the end of the trial (after six months) / follow-up
