# Supplementary material for: Health information management of older, multimorbid patients in German primary care: feasibility and first results of the outcome measures of a cluster-randomised controlled pilot trial – HYPERION-TransCare
Source: BMC Prim Care. 2025 Apr 5;26:98. doi: 10.1186/s12875-025-02774-5 (PMC11971799; doi:10.1186/s12875-025-02774-5)
Supplement: Supplementary file 2 — Additional file 2. Table of contents of the patient portfolio. [file 12875_2025_2774_MOESM2_ESM.pdf]

Appendix B: Table of contents of the patient portfolio

1 Personal information (to be completed by patient)

a. Key patient data sheet

2 Medication (to be completed by GP)

a. Medication plan
   - incl. indication, dosage regimen, specific information, medication administration, information on rare medications (personalized medications, allergies and intolerances)
   - incl. over-the-counter medications, natural remedies, dietary supplements
   - incl. prescriptions from other doctors in charge of treatment/care providers

b. Insulin plan, if applicable

3 Information on illness(es) (to be completed by GP)

a. Chronic illnesses or conditions
b. Major operations and hospital stays to date
c. Major existing infectious diseases (e.g. hepatitis B+C, tuberculosis, HIV, Covid-19)
d. Examination results and doctor‘s reports (e.g. lab results, images, reports) - GP
e. Examination results and doctor‘s reports (e.g. lab results, images, reports) of other treating physicians

4 Documents (to be completed by GP)

a. Living will
b. Health care proxy
c. Designated power of attorney/guardianship (order by court)
d. Organ donor card
e. Vaccination passport/health insurance card or vaccination status
f. Medication passport, medical device (implant/pacemaker, etc.) ID card
g. Allergy passport
h. Disability card
i. Personal ID card

5 Notes (to be completed by GP and patient)

a. Care-relevant information (e.g. diet, incontinence, obesity, decubitus (tendency), chronic wounds, multi-resistant pathogens) – if applicable, transfer of care form
b. Other allergies and intolerances (e.g. food, bandages, etc.)

6 Hospital preparations (to be completed by GP)

a. Referral form
b. Transport certificate (return transport certificate if necessary)
c. If applicable, any information on what medications must be discontinued

7 Miscellaneous

a. Notepad
b. Empty plastic sleeves for storing documents, blank key patient data sheet

Disclaimer: This is a translation of the original German document and was created for illustrative purposes only. The formatting may not match the original.
