# Supplementary material for: Health information management of older, multimorbid patients in German primary care: feasibility and first results of the outcome measures of a cluster-randomised controlled pilot trial – HYPERION-TransCare
Source: BMC Prim Care. 2025 Apr 5;26:98. doi: 10.1186/s12875-025-02774-5 (PMC11971799; doi:10.1186/s12875-025-02774-5)
Supplement: Supplementary file 3 — Additional file 3. ‘Key patient data’ as part of the patient portfolio. [file 12875_2025_2774_MOESM3_ESM.pdf]

Key patient data#

HYPERION-TransCare SaxoForN
Forschungspraxennetz Allgemeinmedizin Dresden/Frankfurt am Main

Last name, first name				
Date of birth			Interpreter required<br>☐ No ☐ Yes	Language
Address				
Telephone no.			Health insurance provider	
Living arrangement	☐ Partner/ Family	☐ Living alone	☐ Other	☐ Amb. care service
Required level of care established	☐ No	☐ Yes	Level of care:	
Important medications (e.g. emergency or permanent medication) *(see 2)*	☐ Blood thinner (passport)<br>☐ Medication plan is in the portfolio.		Additional:<br>………………………………………………<br>………………………………………………	
Important allergies, medication intolerances *(see 2)*	……………………………………………………………………………………<br>……………………………………………………………………………………			
Chronic illnesses *(see 3)*	……………………………………………………………………………………<br>……………………………………………………………………………………			
Living will *(see 4)*	☐ Yes	☐ No	☐ No resuscitation request<br>☐ No maximum therapy	
Health care proxy *(see 4)*	☐ Yes	☐ No	Designated power of attorney/guardian (name, phone no.)	
Vaccination *(see 4)*	☐ Tetanus		Year: ……………………	
Necessary aids	……………………………………………………………………………………<br>……………………………………………………………………………………			
Special notes (e.g. pacemaker/implants etc.) *(see 4)*	……………………………………………………………………………………<br>……………………………………………………………………………………			

#Disclaimer: This is a translation of the original German document and was created for illustrative purposes only. The formatting may not match the original.

Institut für Allgemeinmedizin Johann Wolfgang Goethe-Universität, Frankfurt am Main
TECHNISCHE UNIVERSITÄT DRESDEN
UNIVERSITÄTS KLINIKUM HEIDELBERG
Was hab' ich?
GEFÖRDERT VOM Bundesministerium für Bildung und Forschung

Key patient data#

HYPERION-TransCare SaxoForN Forschungspraxennetz Allgemeinmedizin Dresden/Frankfurt am Main

Emergency contact	
Name	Relationship to patient
Telephone no.	E-mail address
Name	Relationship to patient
Telephone no.	E-mail address
**Doctors in charge of treatment**	
GP (name)	Practice stamp
Additional doctor (name)	Practice stamp
Additional doctor (name)	Practice stamp
Additional doctor (name)	Practice stamp

Date last updated:	Date last updated: ....
Date last updated:	Date last updated: ....

Reminder to patients: Please keep this key patient data sheet current and carry a copy of it with you in case of emergency. Please inform your emergency contact of the location of your patient portfolio.

Reminder to doctors in charge of treatment: This key patient data sheet is accompanied by a detailed patient portfolio containing important personal health-related documents and information. The emergency contact is informed of the location of the portfolio.

#Disclaimer: This is a translation of the original German document and was created for illustrative purposes only. The formatting may not match the original.

Institut für Allgemeinmedizin Johann Wolfgang Goethe-Universität, Frankfurt am Main
TECHNISCHE UNIVERSITÄT DRESDEN
Carl Gustav Carus 1789 – 1869
UNIVERSITÄTS KLINIKUM HEIDELBERG
Was hab' ich?
GEFÖRDERT VOM Bundesministerium für Bildung und Forschung
