# Supplementary material for: Health information management of older, multimorbid patients in German primary care: feasibility and first results of the outcome measures of a cluster-randomised controlled pilot trial – HYPERION-TransCare
Source: BMC Prim Care. 2025 Apr 5;26:98. doi: 10.1186/s12875-025-02774-5 (PMC11971799; doi:10.1186/s12875-025-02774-5)
Supplement: Supplementary file 4 — Additional file 4. Accompanying GP practice checklist. [file 12875_2025_2774_MOESM4_ESM.pdf]

GP Checklist - Patient Portfolio#

HYPERION-TransCare
SaxoForN
Forschungspraxennetz Allgemeinmedizin Dresden/Frankfurt am Main

☐: Please complete ○: Please remind patient/relatives
🔍: Please check regularly* ✍: Enter your own notes if necessary

Patient's practice no.: ____________________

Patient name: __________________________

General information and documents

Personal information

- ○ Master data sheet (name, address + contact (patient), social history + contact details (incl. caretaker/guardian if applicable), contact details of other important doctors/care providers)
- ☐ GP practice to complete master data sheet if necessary
- ☐ Patient preferences (resuscitation request, maximum therapy) worked out together

✍ ____

Medication

- ☐ Current medication plan (+ insulin plan, if applicable), incl. indication, medication administration schedule + specific information, application + allergies and intolerances
- ○ Supplements: over-the-counter medications, natural remedies, dietary supplements, prescriptions from other doctors/care providers
- ☐ Note: rare medications (e.g. personalised medications)

✍ ____

Illness-specific information

- ☐ Permanent diagnoses (not only ICD code), relevant previous diagnoses
- ☐ Relevant operations and hospital stays
- ☐ Existing infectious diseases / multi-resistant pathogens (e.g. Hep B+C, TB, HIV)
- ☐ Most recent lab results, older lab results for comparison if necessary
- ☐ Currently applicable previous findings on file (e.g. ultrasound, etc.)
- ○ Currently applicable external previous findings (e.g. letters, printouts, images, etc.)

✍ ____

Documents

- ○ Copy of ID card
- ○ Organ donor card
- ○ Disability card
- ○ Allergy passport
- ○ Vaccination passport (status) / health insurance card
- ○ Living will, health care proxy, designated power of attorney/guardianship (order by court)
- ○ Medication passport, medical device (implant/pacemaker, etc.) ID card

✍ ____

#Disclaimer: This is a translation of the original German document and was created for illustrative purposes only. The formatting may not match the original.

Institut für Allgemeinmedizin
Johann Wolfgang Goethe-Universität, Frankfurt am Main
TECHNISCHE UNIVERSITÄT DRESDEN
UNIVERSITÄTS KLINIKUM HEIDELBERG
GEFÖRDERT VOM
Bundesministerium für Bildung und Forschung

Specific information + reminders

- ▀ If applicable, attach the transfer of care form master data sheet (excl. medication & diagnoses).
- ▀ Health status/function level/cognitive assessment (e.g. Barthel Index)
- ▀ Care-relevant information (e.g. diet, incontinence, obesity per magna, decubitus (tendency), chronic wounds)
- ○ Other allergies and intolerances (e.g. food, bandages, etc.)
- ○ Document any language or interpreter requirements if applicable.
- ✍ ______________________________

<u>Referral-specific information and documents</u>

Organizational preparation for hospital stay

- ▀ Referral form + information on the reason for referral (if no other referrer)
- ▀ Transport certificate (also return transport certificate if necessary)
- ▀ Any necessary discontinuation of medication
- ▀ If applicable, information to nursing service staff and obtaining a transfer of care form
- ○ Reminder of health insurance card + personal health folder (complete?)
- ○ Reminder of any necessary aids (e.g. CPAP machine, rollator)
- ▀ Rare medications (e.g. personalised medications): Reminder for patient to bring along prescription or have a new prescription issued if necessary

Date last processed	Signature	Date last processed	Signature

Your notes:

____

____

____

____

*Regularly= if there is a change, but check at least once a quarter

[#]Disclaimer: This is a translation of the original German document and was created for illustrative purposes only. The formatting may not match the original.

Institut für Allgemeinmedizin
Johann Wolfgang Goethe-Universität, Frankfurt am Main
TECHNISCHE UNIVERSITÄT DRESDEN
Carl Gustav Carus 1789 - 1869
UNIVERSITÄTSKLINIKUM HEIDELBERG
GEFÖRDERT VOM
Bundesministerium für Bildung und Forschung
