# Supplementary material for: Health information management of older, multimorbid patients in German primary care: feasibility and first results of the outcome measures of a cluster-randomised controlled pilot trial – HYPERION-TransCare
Source: BMC Prim Care. 2025 Apr 5;26:98. doi: 10.1186/s12875-025-02774-5 (PMC11971799; doi:10.1186/s12875-025-02774-5)
Supplement: Supplementary file 5 — Additional file 5. Comparison of HYPERION-TransCare physician characteristics with NASHIP data. [file 12875_2025_2774_MOESM5_ESM.pdf]

Table Appendix E: Comparison of HYPERION-TransCare physician characteristics with NASHIP* data

	NASHIP*	HYPERION-TransCare GPs
Age in %		
under 40 years	7.3	16.7
40-49 years	21.8	25.0
50-59 years	34.7	25.0
60-65 years	20.5	25.0
over 65 years	15.6	8.3
Sex in %		
female (%)	48.8	33.3
male (%)	51.2	66.7
Types of practices in %		
Single practice	54	50
Group practice by partnership agreement (*Berufsausübungsgemeinschaft* - BAG)	39	50
Ambulatory heathcare center/facility (*Medizinisches Versorgungszentrum* - MVZ)	7	0

* GP specialty groups from the Federal Register of Physicians of the National Association of Statutory Health Insurance Physicians (NASHIP) from 2021
